# Neovaginal Perforation in Sigmoid Vaginoplasty: An Underrecognized Complication—A Literature Review

**DOI:** 10.3390/medicina61040691

**Published:** 2025-04-09

**Authors:** Yen-Ning Huang, Jeng-Fu You, Ching-Hsuan Hu

**Affiliations:** 1 Department of Plastic and Reconstructive Surgery, Chang Gung Memorial Hospital, Chang Gung Medical College and Chang Gung University, Taoyuan 333, Taiwan; tim.0egghead0@gmail.com; 2 Department of Colon and Rectal Surgery, Chang Gung Memorial Hospital, Chang Gung Medical College and Chang Gung University, Taoyuan 333, Taiwan; jenodyssey@gmail.com

**Keywords:** sigmoid vaginoplasty, neovaginal perforation, gender affirmation surgery, surgical complications, transgender health, postoperative care

## Abstract

*Background and Objectives*: Gender affirmation surgery significantly improves the quality of life and psychological well-being of transgender women. Among various techniques, sigmoid vaginoplasty is widely performed due to its ability to provide adequate vaginal depth and intrinsic lubrication. However, it carries risks, with neovaginal perforation being a serious yet underreported complication. *Materials and Methods*: This review examines the etiology, clinical manifestations, diagnosis, and management of neovaginal perforation. A literature review was conducted to analyze reported cases and treatment strategies. Additionally, we present a case from our institution to highlight diagnostic and therapeutic challenges. *Results*: Neovaginal perforation arises from mechanical trauma, ischemia, infection, or structural weaknesses in the sigmoid segment. Common risk factors include improper dilation, introital stenosis, and vascular compromise. Symptoms range from mild pelvic discomfort to peritonitis and sepsis. Computed tomography (CT) is the gold standard for diagnosis. Conservative management is effective in mild cases, whereas severe cases require surgical repair. *Conclusions*: Neovaginal perforation is rare but potentially life-threatening. Future research should refine surgical techniques, dilation protocols, and tissue engineering solutions. Standardized guidelines and patient education are essential for prevention and improved outcomes.

## 1. Introduction

Gender affirmation surgery is a critical component of the transition process for transgender women, significantly enhancing their quality of life and psychological well-being. As surgical expertise continues to expand, a variety of techniques have been developed, with penile inversion, peritoneal vaginoplasty, and sigmoid vaginoplasty being the most commonly performed procedures [1]. The choice among these techniques depends on multiple factors, including surgical invasiveness, available penile skin, vaginal depth, aesthetic outcomes, and functional properties.

Sigmoid vaginoplasty, also known as rectosigmoid neocolporrhaphy or neocolpopoiesis, was first conceptualized by Baldwin in 1904, utilizing an intestinal segment for vaginal reconstruction [2,3]. Building on this approach, Wallace successfully employed the sigmoid colon in 1911 [4]. The technique was later introduced for gender affirmation surgery by Markland and Hastings and has since gained widespread adoption [5]. With advancements in open, laparoscopic, and robotic-assisted approaches, modern sigmoid vaginoplasty now offers enhanced visualization, minimal invasiveness, superior cosmetic outcomes, and faster recovery times [1,6,7].

One of the key advantages of sigmoid colon vaginoplasty is its ability to provide sufficient vaginal depth and width, along with mucosal secretion that facilitates lubrication and reduces postoperative shrinkage [3,8,9]. However, despite these benefits, it remains a technically complex procedure requiring multidisciplinary collaboration and carries a risk of postoperative complications. The most frequently reported issues include introital stenosis and mucosal prolapse, while rarer but more serious complications, such as neovaginal perforation, rectovaginal fistula, and colonic necrosis, have also been documented [1,10,11,12,13,14]. Among these, neovaginal perforation is particularly concerning due to its potential to cause peritonitis, sepsis, and other life-threatening sequelae.

Given its low incidence but high clinical significance, a thorough understanding of the mechanisms, risk factors, and management strategies for neovaginal perforation is essential. This review provides a comprehensive analysis of this underrecognized complication, summarizing the existing literature on its incidence, pathophysiology, and treatment. Additionally, we present a representative case from our institution, offering further insights into the diagnostic and therapeutic challenges associated with this condition.

## 2. Material and Methods

A comprehensive literature search of the PubMed, Scopus, and Google Scholar databases was conducted for studies published between January 2010 and December 2024 for vaginoplasty complication related to neovagina perforation. The Medical Subject Heading terms “vaginoplasty” OR “sigmoid vaginoplasty” OR “neovagina perforation” “complication of sex reassignment surgery” OR “complication of Genital Gender Affirming Surgery” were queried, along with similar derivations of each term. We included case reports and case series that described neovaginal perforation, regardless of the underlying diagnosis (e.g., gender-affirming surgery or MRKH syndrome) or surgical technique. Conference articles or complications not associated with neovagina perforation were excluded. Articles were excluded if they lacked sufficient clinical information, were duplicate reports, or did not specifically address perforation as a complication. No restrictions were placed on publication date or language.

## 3. Result

To date, only case reports have been published, documenting five instances of sigmoid neovaginal perforation, one case of penoscrotal neovaginal perforation, and two cases involving neovaginas in patients with Mayer–Rokitansky–Küster–Hauser (MRKH) syndrome. Several reports have addressed the etiology and potential pathogenic mechanisms.

### 3.1. Etiology and Pathophysiology

The pathophysiology of neovaginal perforation is complex and multifactorial, involving a combination of mechanical trauma, ischemic injury, infection, and structural weaknesses inherent to the sigmoid colon tissue.

**Mechanical Trauma:** One of the most common causes of neovaginal perforation is mechanical trauma, often resulting from improper dilation techniques or vigorous sexual activity [15,16,17]. Postoperative dilation is essential for maintaining vaginal patency, but if performed incorrectly, it can create mucosal tears that compromise the neovaginal wall. These microtraumas may progressively weaken the tissue, leading to deeper submucosal injuries and eventual perforation. Iatrogenic causes should also be considered, particularly in patients undergoing neovaginal dilation or instrumentation. Similarly, excessive force during sexual intercourse, particularly in the early postoperative period, may contribute to structural failure [16,18].

**Ischemia and Vascular Compromise:** Vascular integrity plays a crucial role in neovaginal viability. If the sigmoid segment used for neovaginal construction experiences inadequate perfusion due to anastomotic disruption, arterial insufficiency, or venous congestion, ischemic necrosis can develop. This can lead to ulceration and thinning of the neovaginal wall, predisposing it to perforation. Introital stenosis may also exacerbate this issue by restricting blood flow, further increasing the risk of tissue breakdown [1].

**Infection and Abscess Formation:** Neovaginal perforation can be further complicated by bacterial infections, particularly when colonic bacteria invade the surrounding pelvic or peritoneal structures. Infections may originate from minor mucosal defects, which serve as entry points for bacterial translocation. If untreated, these infections may progress to abscess formation or sepsis, necessitating urgent surgical intervention [18].

**Postoperative Complications**: Ischemia or inadequate healing of the bowel anastomosis may also predispose patients to neovaginal perforation. In cases where compromised blood supply leads to ischemic necrosis of the neovaginal wall, the structural integrity of the tissue is weakened, rendering it more vulnerable to rupture. Infection, another well-recognized complication following intestinal-based neovaginoplasty, can further contribute to tissue degradation and increase the risk of perforation [1].

**Introital Stenosis:** Introital stenosis refers to a narrowing of the neovaginal introitus that may cause difficulty with dilation, sexual activity, or neovaginal drainage [11,19,20]. Introital stenosis is a common postoperative complication in neovaginal reconstructions. In patients with neovaginal stenosis, increased intravaginal pressure during dilation or intercourse may exert excessive force on the weakened neovaginal wall, increasing the likelihood of perforation. Additionally, inadequate postoperative care and failure to adhere to dilation protocols may contribute to the progressive narrowing of the neovaginal introitus, thereby exacerbating the risk [18,19].

A systematic analysis of potential risk factors for neovaginal perforation is summarized in Table 1.

### 3.2. Clinical Presentation and Case Reports

Neovaginal perforation following sigmoid vaginoplasty can present with a wide range of clinical manifestations, depending on the severity and timing of the perforation.

In the early stages, symptoms may be mild, including vague pelvic discomfort, localized tenderness, or abnormal vaginal discharge. As the perforation progresses, more severe signs of infection, such as fever, nausea, vomiting, and systemic inflammatory response syndrome (SIRS), may develop. If peritonitis occurs, patients may exhibit severe abdominal pain, rebound tenderness, and signs of sepsis, including hypotension and tachycardia.

A significant proportion of reported cases highlight that introital stenosis plays a critical role in neovaginal perforation. In these cases, patients often experience increasing difficulty with dilation, followed by a sudden inability to insert the dilator fully. This may be accompanied by deep-seated pain within the neovagina, which is often mistaken for simple stenosis. However, continued forceful dilation in the presence of underlying ischemia or tissue fragility can lead to catastrophic rupture.

### 3.3. Reported Cases

Several case reports related to sigmoid neovagina perforation illustrate the diverse presentations and management strategies for neovaginal perforation (Table 2).

- **Liguori et al. (2001)** documented a case of acute peritonitis secondary to introital stenosis, leading to perforation of a neovagina constructed from a bowel segment [19].
- **Amirian et al. (2011)** reported a patient who presented with lower abdominal pain and fever [21]. CT imaging revealed free air in the retroperitoneum, and a leak through the vaginal apex was confirmed via vaginal contrast examination. This patient was successfully managed with conservative antibiotic therapy.
- **Shimamura et al. (2015)** described a case of neovaginal perforation complicated by an intra-abdominal abscess, where the clinical symptoms and radiologic findings were incongruent [18]. Surgical intraperitoneal drainage was performed due to concerns that the abscess might not resolve with antibiotics alone.
- **Meece et al.** reported two cases involving diffuse stenosis of unknown etiology, leading to ischemia and subsequent perforation of the sigmoid conduit [1]. One patient underwent midline laparotomy and was found to have multiple interloop abscesses, requiring prolonged intravenous antibiotic therapy. The second patient, who developed vaginal stenosis secondary to a high-riding perineum, required laparoscopic sigmoid conduit resection, followed by a midline incision and internal suturing of the colon flap one month postoperatively.

**Figure 1:**
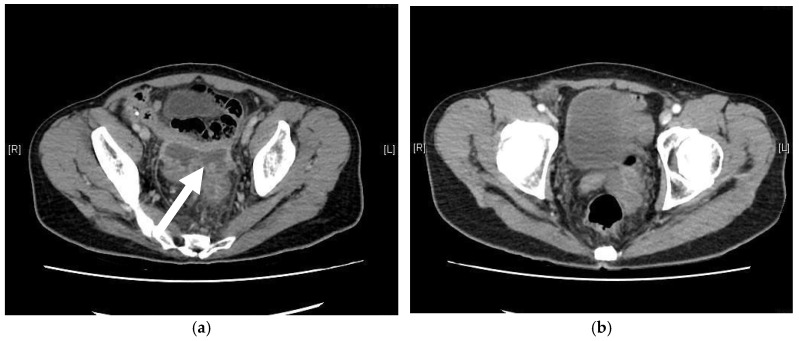

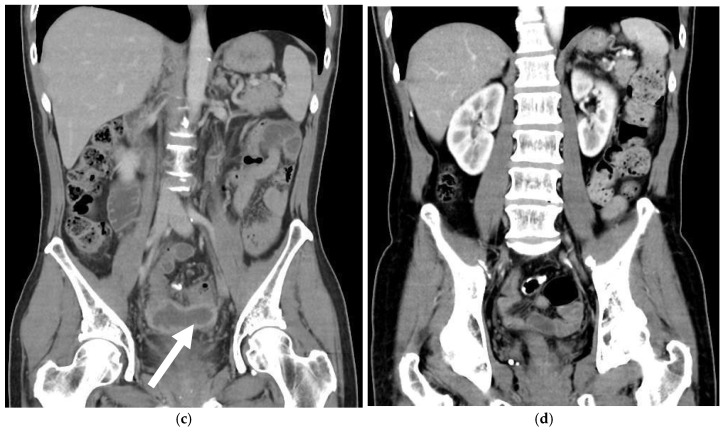
Serial contrast-enhanced abdominal CT images demonstrating the clinical course of an intra-abdominal abscess following sigmoid neovaginoplasty. (**a**) Axial contrast-enhanced CT image obtained at initial presentation reveals a large intra-abdominal abscess occupying a significant portion of the pelvic cavity. The abscess is well circumscribed with rim enhancement, indicating mature, encapsulated fluid collection. The white arrows highlight the abscess cavity abutting the neovaginal wall, suggestive of potential neovaginal origin or involvement. (**b**) Axial CT image obtained six months after appropriate surgical and medical management shows complete resolution of the abscess. The neovagina appears patent, without evidence of perforation, surrounding inflammation, or fluid re-accumulation. (**c**) Sagittal view of the abdomen on contrast-enhanced CT at the time of diagnosis demonstrates the same encapsulated intra-abdominal abscess located in the rectovaginal space. The fluid collection is again well circumscribed with an enhancing capsule, as indicated by the white arrow, compressing adjacent structures. (**d**) Follow-up sagittal CT image six months post-treatment confirms resolution of the abscess. The neovagina remains intact and clearly delineated, with no signs of perforation or residual infection, indicating successful treatment and structural preservation.

**Figure 2:**
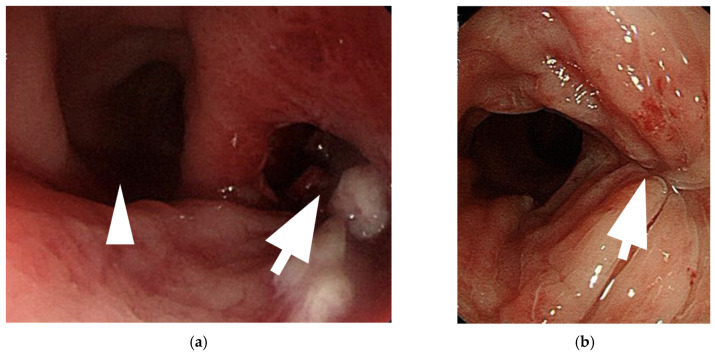
Colonoscopic evaluation of neovaginal perforation and post-treatment healing. (**a**) Initial colonoscopic image reveals a discrete perforation site located approximately 10 cm proximal to the neovaginal introitus, in the 4–5 o’clock position. The defect is identified on the right-hand side of the neovaginal lumen and is clearly delineated by the white arrow. The neovaginal wall on the left-hand side is visible and indicated by the white arrowhead. Surrounding mucosa appeared erythematous but intact, consistent with a localized perforation without diffuse necrosis. (**b**) Follow-up colonoscopy performed three months after treatment demonstrates complete mucosal healing at the site of the previous perforation. The healed area is visible on the right-hand side, marked by the white arrows, with no evidence of residual perforation, ulceration, or ongoing inflammation. The neovagina appears patent and structurally preserved, confirming effective resolution of the complication.

In addition to these reports, we present a case from our institution, where a 59-year-old transgender woman developed neovaginal perforation following improper dilation. The patient exhibited progressive lower abdominal pain and was found to have an abscess formation above the neovagina (Figure 1). Imaging confirmed gaseous distension and proximal small bowel dilatation. Colonoscopy revealed a mucosal defect at 10 cm above the vaginal introitus. This patient received open drainage via colonoscopy, antibiotic therapy, and a structured rehabilitation program, leading to a full recovery. Three months post-discharge, colonoscopy confirmed complete perforation healing. A six-month follow-up CT scan showed a normal neovagina without residual abscess (Figure 2).

### 3.4. Diagnosis

Due to the nonspecific nature of symptoms, a high index of suspicion is required to diagnose neovaginal perforation. **Computed tomography (CT) imaging** is the gold standard diagnostic modality, as it can identify free air in the peritoneal cavity, abscess formation, or other signs of intra-abdominal infection. In some cases, contrast-enhanced imaging may help delineate the exact site of perforation [21].

For patients presenting with vague abdominal pain and a history of sigmoid vaginoplasty, **pelvic examinations** should be performed with caution. In cases where perforation is suspected, a digital rectal or vaginal exam may reveal areas of tenderness; abnormal mucosal defects; or, in rare cases, direct visualization of a perforation [1]. However, forceful probing should be avoided to prevent exacerbating the injury.

### 3.5. Management Strategies

The management of neovaginal perforation depends on the severity of the condition and the extent of intra-abdominal contamination. In cases where the perforation is small and localized, conservative management with broad-spectrum intravenous antibiotics and bowel rest may be sufficient. Close monitoring for signs of worsening infection is essential, and patients should be advised to avoid any activities that could increase intra-abdominal pressure, such as dilation or sexual activity.

For patients with larger perforations, intra-abdominal abscess formation, or signs of peritonitis, surgical intervention is often required. The choice of surgical approach depends on the extent of the damage and the patient’s overall condition. In some cases, laparoscopic or open surgical repair may be performed to close the defect and drain any associated abscesses. If the neovagina is severely compromised, partial or complete reconstruction may be necessary, utilizing additional bowel segments or alternative techniques such as peritoneal or skin graft-based vaginoplasty.

Postoperative care is crucial in preventing recurrence, and patients should be closely followed to ensure proper healing. Long-term surveillance should include regular gynecologic evaluations, imaging if needed, and patient education regarding safe dilation and sexual practices to minimize the risk of re-injury.

## 4. Discussion

Neovaginal perforation is an uncommon but clinically significant complication following sigmoid vaginoplasty. Although sigmoid vaginoplasty is widely regarded as a reliable technique for neovaginal reconstruction due to its favorable functional outcomes—such as adequate depth, intrinsic lubrication, and long-term tissue durability—rare complications like perforation can lead to substantial morbidity [1,5,10,13]. The mechanisms underlying neovaginal perforation are multifactorial and often interrelated. Contributing factors may include local ischemia caused by excessive tension or compromised vascular supply, mechanical trauma from sexual intercourse or instrumentation, and elevated intraluminal pressure due to introital stenosis or infection. In some cases, chronic inflammation or fibrosis may progressively weaken the neovaginal wall, increasing susceptibility to perforation [19]. Iatrogenic injury is another important consideration, particularly during self-dilation or endoscopic procedures. Khoder et al. reported a case involving simultaneous perforation of the neovagina and urinary bladder during postoperative dilation in a patient with Mayer–Rokitansky–Küster–Hauser (MRKH) syndrome [16]. This highlights the critical importance of patient education and careful technique supervision in the postoperative period to minimize such risks.

Alternative techniques for neovaginal reconstruction, such as penile inversion, skin graft-based vaginoplasty, and peritoneal vaginoplasty, have also been described in the literature [22,23,24,25]. These techniques vary in their vascular supply, support structures, and long-term durability. While perforation appears to be more frequently reported in sigmoid-based neovaginas, other techniques are more commonly associated with complications such as stenosis, necrosis, or granulation tissue formation [13]. Long-term functional outcomes of sigmoid vaginoplasty generally include favorable neovaginal depth, mucosal lubrication, and sexual satisfaction. However, complications such as perforation may significantly compromise these outcomes, leading to chronic infection, pain, or fistula formation, and sometimes necessitating surgical revision [16,26].

Even within sigmoid vaginoplasty itself, technical variations—including differences in segment length, mesenteric tension, anastomosis orientation, and fixation to pelvic structures—may influence tissue perfusion and mechanical stability, thereby potentially affecting the risk of neovaginal perforation. Despite these plausible associations, direct comparative studies evaluating technique-specific complication profiles are currently lacking.

Preventive strategies—including meticulous surgical techniques, adequate vascular preservation, tension-free anastomosis, and secure fixation—along with close postoperative monitoring and appropriate dilation protocols, are essential to reduce the risk of perforation. Surgical experience plays a critical role in minimizing complications. Surgeons familiar with neovaginal construction and pelvic reconstruction are more likely to achieve favorable outcomes, particularly when managing complex cases or revisional surgery.

## 5. Future Directions

Although most reported neovaginal perforations are acute and clinically significant, the potential impact of small, subclinical perforations should not be underestimated. It remains unclear whether these minor injuries may predispose patients to delayed complications, such as fistula formation. Chronic micro-injury or undetected breaches in neovaginal integrity may, over time, create a conduit for fistula development—particularly in anatomically complex or surgically altered fields. Considering the delicate nature of neovaginal tissue, especially in graft- or bowel-derived reconstructions, persistent low-grade trauma or unrecognized micro-perforations may plausibly contribute to this process. However, the current literature lacks longitudinal data on the progression and long-term sequelae of such subclinical events, highlighting the need for further investigation.

Given the rarity of neovaginal perforation, there is a clear need for more comprehensive research to better understand its risk factors, prevention strategies, and optimal management approaches. One promising avenue for future studies is tissue engineering and regenerative medicine, which may offer alternative solutions for neovaginal reconstruction that reduce the risk of perforation. Research into bioengineered vaginal grafts, composed of autologous stem cell-derived tissues, may provide a more durable and structurally resilient neovaginal lining compared to traditional bowel-derived techniques.

Subtle variations in surgical technique may play a role in neovaginal perforation risk by influencing tissue perfusion and structural integrity. Although certain technical factors have been hypothesized to contribute, direct comparative evidence remains scarce. To improve outcomes, future research should prioritize prospective data collection, multicenter registries, and standardized complication reporting. In parallel, biomechanical investigations of neovaginal constructs may help refine operative approaches. Additionally, many transgender women may not be fully aware of the specific risks associated with intestinal-based neovaginal reconstruction, including perforation. Developing comprehensive patient education materials and support programs will help ensure informed decision-making and improve postoperative care.

## 6. Limitations

This review is limited by the lack of large-scale or prospective data, as the current analysis relies entirely on published case reports. This inherently introduces a risk of publication bias and limits the generalizability of the findings. Furthermore, there is significant heterogeneity among the reported cases, including variations in surgical techniques, postoperative follow-up duration, and diagnostic criteria. These differences pose challenges in drawing direct comparisons or establishing standardized conclusions. Future studies with standardized data collection and larger cohorts are needed to better understand the clinical patterns and outcomes associated with neovaginal perforation.

## 7. Conclusions

Neovaginal perforation is a rare but potentially serious complication of sigmoid vaginoplasty. While the procedure remains a reliable option for neovaginal reconstruction, patients should be counseled about its risks, including perforation. Mechanical trauma, introital stenosis, and postoperative complications may contribute to its occurrence, though data remain limited. Early recognition, multidisciplinary management, and proper surgical technique are key to reducing morbidity. Further research is needed to clarify the risk factors and optimize prevention strategies, ensuring safe and effective outcomes for transgender women.

## Figures and Tables

**Table 1:** Potential risk factors of neovaginal perforation.

Category	Risk Factor	Potential Impact
**Patient-Related Factors**	MRKH syndrome or prior pelvic anomalies	Anatomical challenges and altered tissue planes may increase susceptibility
	Poor compliance with dilation protocols	Improper dilation may lead to stenosis or pressure-related trauma
	Comorbidities (e.g., diabetes, smoking)	Impaired healing and tissue fragility
**Surgical Factors**	Excessive graft length or tension	Increased mechanical stress and risk of ischemia
	Inadequate neovaginal fixation	Higher risk of graft movement or instability
	Ischemia or poor vascular supply	Tissue necrosis or perforation risk
**Postoperative Factors**	Introital stenosis	Distal obstruction and pressure buildup
	Traumatic intercourse or instrumentation	Direct mechanical trauma to the neovaginal wall
	Inadequate postoperative follow-up	Delayed detection of complications

**Table 2:** Reported cases of neovaginal perforation.

	2001 Liguori et al. [19]	2011 Amirian et al. [21]	2015 Shimamura et al. [18]	2023 Meece et al. [1]	2023 Meece et al. [1]
Early complication after surgery	Total introital stenosis of the neovagina	no	Mild stenosis of the neovagina	Cellulitis and prolonged urinary retention on postoperative days 19 and 20	Vaginal stenosis secondary to a high riding perineum
Symptom	Colicky abdominal pain, abdominal distension, and vomiting	Lower abdominal pain and fever	Persistent abdominal pain, nausea, and vomiting	Abdominal pain, vomiting, fever, large volume of mucinous discharge, and an inability to dilate	Abdominal pain, fever, nausea, vomiting
Onset	1 year postoperatively	Unknown	Unknown	1 year postoperatively	3 years postoperatively
Image findings	A large amount of fetid mucus in the abdominal cavity via laparoscopy	Free air in the retroperitoneum by CT A leak through the vaginal top via vaginal contrast examination	CT: a massive abscess occupying a significant portion of the intra-abdominal cavity	A significant vaginal stricture A 2 cm perforation of the sigmoid conduit (necrotic) Multiple dense adhesions of the small bowel and right colon in the pelvis	Completely occluded neovagina at the phallo-collic anastomosis Sigmoid conduit severely dilated, ischemic, with dense small bowel adhesions at the proximal portion, perforation at the most proximal aspect of the conduit
Management	Exploratory laparotomy with primary repair	Intravenous antibiotics only	Exploratory laparotomy with primary repair	Midline laparotomy and resection of the necrotic sigmoid conduit	Laparoscopic resection of the sigmoid conduit
Prognosis	Recurrent total stenosis of the neovaginal introitus	Fair and no complications noted	No complications related to the surgery	Without further complication	Without further complication

## Data Availability

The original contributions presented in this study are included in this article. Further inquiries can be directed to the corresponding author.
